# Pyrolyzed Photoresist as a Continuous‐Tone Mask for Grayscale Lithography

**DOI:** 10.1002/advs.78394

**Published:** 2026-10-18

**Authors:** Tielyr D. Creason, Gavin C. Gee, Zachary T. Piontkowski, Toai Ton‐That, Robert Reyna, Edward R. Arata, Mason Risley, Jacob D. Boissiere, Bryan Kaehr, D. Bruce Burckel

**Affiliations:** ^1^ Sandia National Laboratories Albuquerque New Mexico USA

**Keywords:** grayscale, lithography, pyrolyzed carbon

## Abstract

Grayscale lithography enables single‐step fabrication of continuous 3D surface relief for complex optics, MEMS, and scalable replication templates. However, its broad adoption is hindered by the cost, complexity, and resolution constraints associated with fabricating high‐quality grayscale masks. Here, we address this challenge by developing a method to produce continuous‐tone photolithography masks/reticles through the pyrolysis of 2.5D photoresist structures fabricated using standard multiphoton lithography systems. During pyrolysis, the pattern densifies from micrometers in thickness in the resist state to nanometers in thickness in the carbon state. While the pyrolyzed patterns undergo significant vertical shrinkage, adhesion to the substrate is maintained, preserving overall lateral pattern fidelity despite a systematic in‐plane expansion. The amorphous carbon is chemically inert and mechanically robust, enabling repeated exposure and mask cleaning steps, converting the single‐use polymer pattern written via direct‐write technology into an optical mask capable of repeated exposures.

## Graphical abstract

True grayscale contact photolithography masks are created by pyrolysis of 3D multiphoton resist patterns. The resulting carbon masks retain micrometer‐scale patterned features while providing continuous‐tone optical transmission. This approach provides access to high‐quality grayscale lithography without requiring exotic materials or commercial mask fabrication for facilities with multiphoton printing capability.

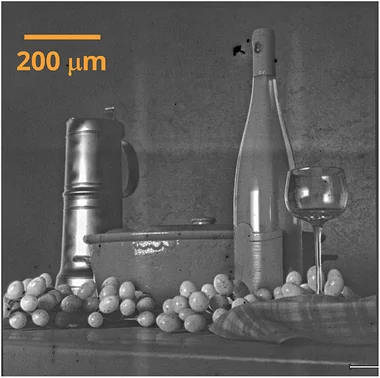

## 1 Introduction

Micro‐lens arrays [1] and diffractive optical elements [2] are examples of non‐planar micrometer‐scale devices which are particularly challenging to fabricate when using semiconductor fabrication techniques designed for integrated circuits. Early research into fabrication of micro‐optics employed resist reflow to create micro‐lens arrays [3]. While resist reflow can create plano‐convex shapes, the reliance on the surface tension of the resist to form curvature makes it incapable of creating arbitrary height profiles. Diffractive optical elements can be fabricated by sequential binary mask exposure/etch steps resulting in a stepwise linear approximation of the continuous phase prescription required for optimal optical performance, but this approach suffers from multiple process steps, alignment/overlay challenges, and lower optical efficiency due to the imperfect approximation of the phase prescription.

Grayscale lithography (GSL) is a fabrication technique that can create the non‐planar topography necessary for micro‐lens arrays and diffractive optical elements. GSL defines arbitrary resist height profiles by allowing partial exposure/development of photoresist, resulting in resist profiles with height determined by the local UV transmission in the mask [4, 5, 6]. However, fabrication of high‐fidelity micrometer‐scale grayscale masks remains technically challenging. One approach is to use a “dithered” half‐tone mask [7], where regions are pixilated on a deep sub‐micrometer scale, and the local transmission of the region is controlled by the ratio of opaque to transparent pixels in the region. This approach requires low‐throughput e‐beam lithography to create the deep sub‐micron pixels and results in limited grayscale contrast as the pixilation averaging approach breaks down for extremely sparse (clear) and dense (opaque) regions. Another approach for creation of grayscale masks involves using high‐energy beam sensitive (HEBS) glass [8], again, patterned using an e‐beam system. The HEBS‐glass approach requires use of a bespoke glass composition with a limited number of suppliers/longer lead times and thus ill‐suited for optimization/prototyping. There are also more recent continuous grayscale mask approaches such as thermochemical conversion of CrOx films [9] and direct laser write conversion of Sn films [10]. Finally, there are more exotic solutions [11, 12] for grayscale mask fabrication, which all offer varying advantages and drawbacks.

The last decade has witnessed a boom in lithographic techniques that use direct write, non‐linear photo‐physical absorption such as multiphoton lithography (MPL) [13, 14] or two‐step absorption [15]. These approaches depart from the conventional planar fabrication approach and enable direct creation of three‐dimensional structures with complex nano/microscale features [16], opening new design paradigms in microfabrication that are not possible with planar lithography approaches. Despite recent advances to improve throughput [17, 18], at large, these approaches are single‐use patterns and thus incompatible to rapid prototyping. Reducing the cost and turnaround time of grayscale mask fabrication can make continuous‐relief patterning more accessible for rapid prototyping and design optimization in micro‐optics, MEMS, microfluidics, surface wettability engineering, biomimetic texturing, structured adhesives, and scalable replication templates.

To these ends, we introduce Grayscale Lithography by Annealed Resin Engineering (GLARE), a grayscale masking strategy based on pyrolyzed carbon films with thickness‐dependent optical attenuation. We demonstrate that transparent, MPL‐written‐2.5D polymer patterns with micrometer‐scale thickness pyrolyze to opaque, nanometer‐thick carbon structures that produce grayscale exposure when used as a mask for contact lithography. Despite considerable volumetric shrinkage, lateral pattern fidelity is largely maintained, with only a small systematic in‐plane expansion, yielding smooth glassy carbon surfaces as confirmed by AFM and Raman characterization. We observe that the transmission of the carbon mask at 365 nm varies systematically with carbon thickness, enabling an empirical relationship between amorphous carbon thickness and i‐line (365 nm) transmission to be established. The approach was applied to fabricate a functional microlens array, demonstrating its applicability to grayscale micro‐optical structures. The reusability of the masks is examined using standard micro‐fab cleaning processes, resulting in no discernable degradation, supporting their potential implementation in scalable manufacturing environments.

## 2 Results

### 2.1 Concept and Methodology for Grayscale Mask Fabrication

Pyrolysis is the thermochemical decomposition of a material at elevated temperature in the absence of oxygen, which transforms the material into a more carbonized, ceramic, or otherwise chemically altered state. Previous studies have leveraged pyrolysis of MPL‐fabricated polymer precursors to produce polymer‐derived ceramic microstructures such as SiOC, SiC, and Si_3_N_4_, demonstrating that complex three‐dimensional architectures can be retained through conversion to high‐performance ceramic materials [19, 20]. Applying this to MPL carbon‐rich photoresins results in conversion to glassy carbon [21, 22, 23, 24, 25], a material characterized by high chemical and thermal resistance [26]. Recognizing the thickness‐dependent optical opacity of pyrolyzed carbon films [27, 28, 29] and the ability of MPL to generate complex 2.5D topographies, we developed a scalable strategy for fabrication of continuous‐tone grayscale masks.

Figure 1a shows a schematic diagram of the process used to create the GLARE masks. An image profile is converted into a digital grayscale image (e.g., bmp, png), which serves as a height map for a scanning laser MPL system (Nanoscribe Quantum X). The resultant topographic grayscale replica is rendered in photoresin (IP‐S, a negative‐tone photoresist for from Nanoscribe GmbH) onto a transparent substrate suitable for high‐temperature conversion (e.g., fused silica or quartz). The patterned substrate is then pyrolyzed in a reducing atmosphere. The non‐carbon species in the resist evaporates or sublimes off while the carbon remains, yielding an amorphous glassy carbon replica of the digital image. The mask blank can then be used as a contact mask to perform grayscale contact lithography to create grayscale resist patterns in photoresist in a single lithographic exposure taking seconds versus than the serial process used to write the MPL pattern (hours to days depending on the areal size and complexity). Figure 1b illustrates this workflow using a grayscale digital image of a human eye. The source image is converted to a spatial mapping with each pixel representing 1 µm. The 0–255 grayscale of the image pixels are converted to a z‐height, mapped from 0 (black) to 8 µm (white) in GrayScribeX (IP‐S default settings) and written with the Quantum X using two‐photon grayscale lithography (2GL). Following pyrolysis, the resultant contact mask recreates the image in positive‐tone photoresist.

**FIGURE 1:**
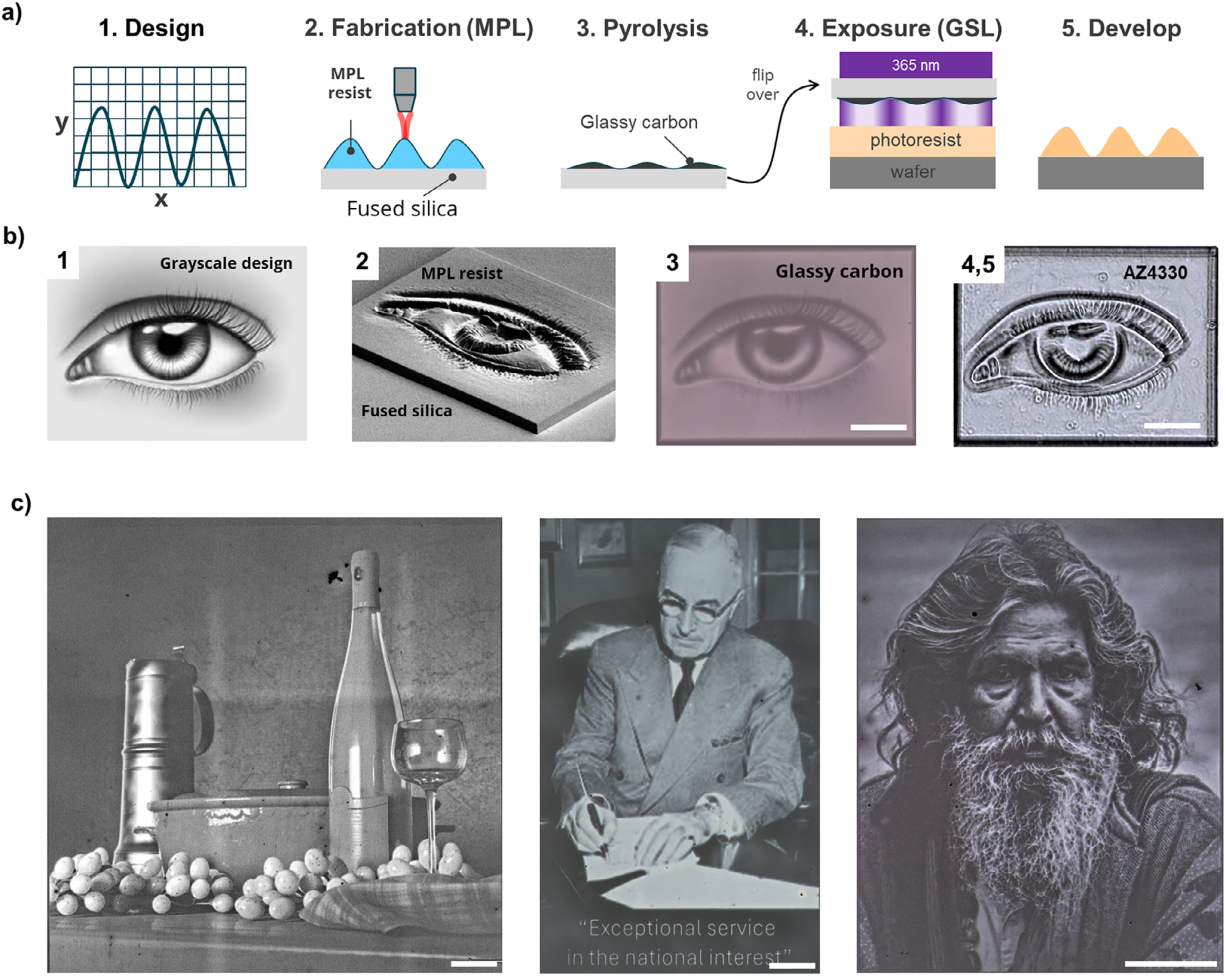
Grayscale Lithography by Annealed Resin Engineering (GLARE). (a) Schematic showing a 5‐step process for creation and application of grayscale masks; (b) Grayscale image and resultant patterned materials created from the GLARE process. Scale bars, 50 µm. (c) Optical microscopy images of images rendered into grayscale lithography masks (left panel image courtesy of Catherine De La Torre [30]; center panel image courtesy of the U.S. Department of Defense, public domain; right panel image from subhamshome, Pixabay (2017), under the Pixabay Content License). Scale bars, 100 µm.

### 2.2 Characterization of Pyrolyzed Photoresist on Fused Silica

#### 2.2.1 Image Preservation, Shrinkage and Adhesion

Thermogravimetric analysis of organic resists during pyrolysis indicates mass loss due to volatilization of non‐carbon species completes at approximately 600°C, while other physical properties of the film such as conductivity continue to evolve after heating to greater than 800°C [31]. For this reason, a pyrolyzation temperature above this threshold was chosen (900°C), which was used in previous work to convert micrometer‐scale interferometric‐defined 3D resist patterns into carbon scaffolds [32]. These scaffolds were found to compatible with harsh solvents while maintaining adhesion to substrates [22, 33, 34]. Figure 1c shows optical microscope images of fused silica substrates after pyrolysis illustrating the fine details of the images are maintained.

Fabrication of a deployable grayscale mask requires precise knowledge of the relationship between the thickness of the initial resist structure, the thickness of the carbon replica, and the transmission at the exposure wavelength through the mask. To understand volumetric shrinkage and potential image distortion, MPL was used to write a structure with a sinusoidal variation in resist height. Figure 2a (upper panel) shows a false‐color laser profilometer image (Keyence VK‐X3100), while the upper inset shows a tilted scanning electron micrograph (SEM) of the structure. Figure 2a (lower panel) contains a white‐light interferometer (Keyence VK‐X3100) measurement of the resulting carbon structure after pyrolysis. The inset shows an SEM image of the same structure after pyrolysis. Note the change in *z*‐axis scale between the upper and lower panels of **2a**, where the vertical (height) axis changes by more than 2 orders of magnitude. To quantify the pattern fidelity of the pyrolyzed resist, traces of the sinusoidal topography were measured and normalized to the maximum height. Figure 2b shows a graph of the normalized heights for the as‐printed (black line) and post‐pyrolyzed (red line) data. While the initial printed sine wave displays uniform peak heights, the subsequent pyrolyzed structures exhibit slight variations in peak height, albeit only on the nanometer scale. Resolving the nanometer scale of the pyrolyzed sample also exposes the lack of flatness of the substrate (excursion below 0 on the graph around 25 micrometers on the horizontal axis). The period of the carbon sinusoid appears slightly stretched compared to the pre‐pyrolyzed version (exhibiting a mean absolute peak position deviation of ∼2.0 µm relative to the printed master), which may be due to minor distortion of the pattern during conversion to glassy carbon or plastic deformation of the substrate, which can occur at elevated temperatures (> 600 C [35]).

**FIGURE 2:**
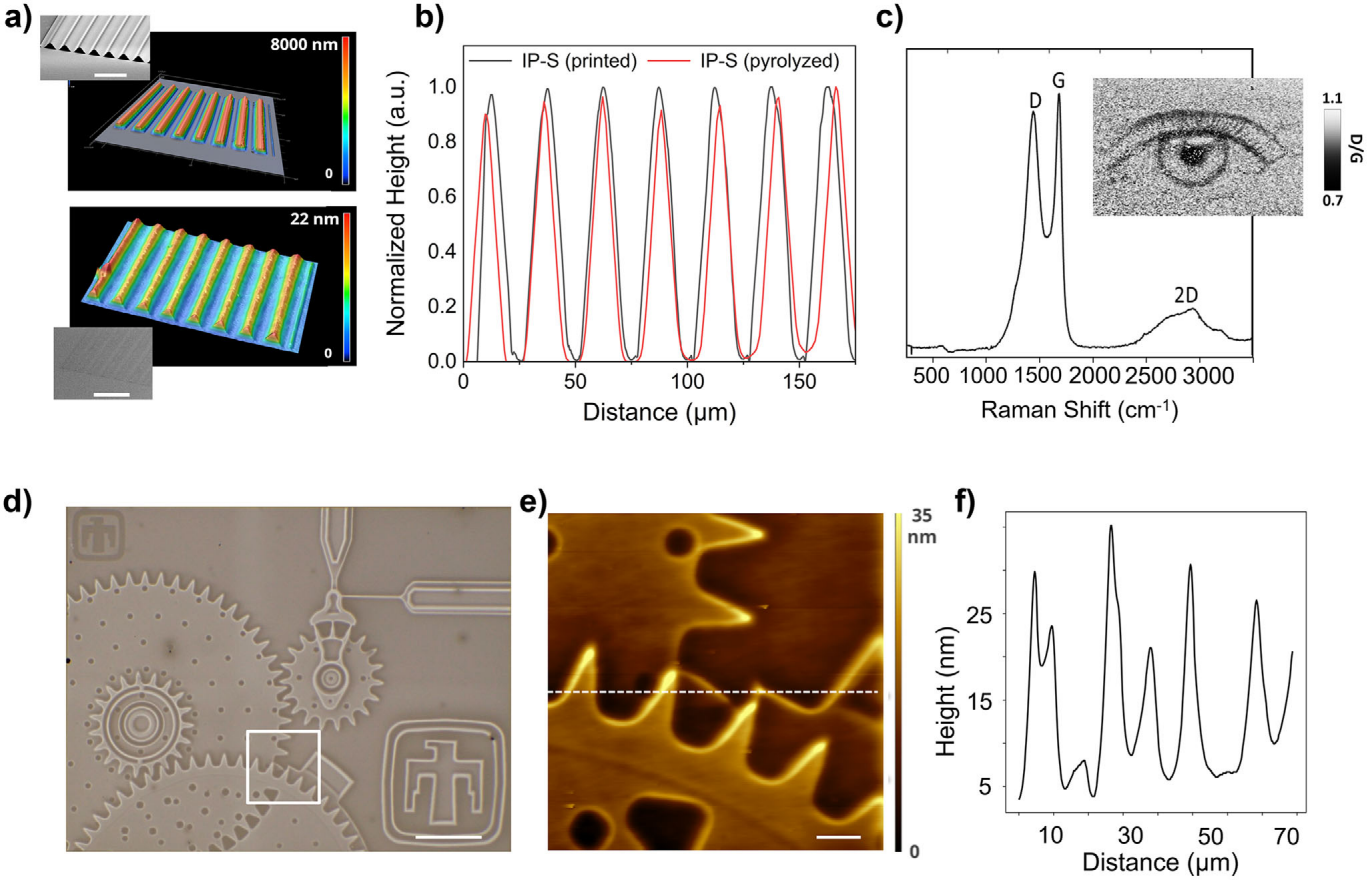
Structural characterization of GLARE masks. (a) Profilometer scans of MPL printed sinusoidal pattern before (top panel) and after pyrolysis (bottom panel; white‐light interferometer scan). Insets show SEM images of the pattern (scale bars, 50 µm). (b) Overlaid graphs of the normalized resist and carbon sinusoids indicate low distortion. (c) Raman scan of the carbon mask indicating disordered/low‐crystallinity carbon; Inset shows the image created by mapping the D‐peak and dividing by the G‐peak map to produce a D/G ratio indicative of pyrolytic/glassy carbon. (d) Optical microscope image of a GLARE mask created from an SEM image of MEMs gears; (e) AFM image of the region outlined in the white square in (d); (f) Graph of a profile taken through the dotted line AFM image in (e).

Table S1 indicates the volumetric shrinkage for the IP‐S resist is greater than 99%, which is consistent with prior work pyrolyzing IP‐series resist to make suspended carbon AFM tips [24]. Yet, the printed image and image contrast are maintained, with only a modest systematic change in lateral dimensions (∼2%–4% expansion). Because this dimensional change is systematic, it's possible to account for it by pre‐scaling the written design when dimensional fidelity is critical. This disparity between vertical and lateral shrinkage during resist pyrolysis is a known phenomenon, with previous studies attributing this anisotropic shrinkage behavior primarily to: 1) adhesion to the substrate, and 2) larger exposed surface area in the vertical direction [25]. Considering the former, previous studies of carbon–silica and silicon oxycarbide systems suggest that pyrolysis can promote formation of covalent Si–O–C interfacial species, which likely contribute to this robust adhesion of pyrolyzed carbon films on fused silica substrates [36]. As this bonded adhesion is dependent on processes conditions [31], using a ramp rate of 1°C/min to 900°C (3% H_2_ in Ar) resulted in no delamination of the carbon masks in any of our experiments.

#### 2.2.2 Spectroscopy of Pyrolyzed Films

To understand the structure of the residual carbon, we characterized a variety of grayscale patterns. Figure 2c shows an averaged Raman spectrum of a pyrolyzed region, exhibiting D and G peaks, along with a broad 2D peak, all typical features of disordered carbon [37]. The presence of broad D and 2D disorder peaks rules out large‐scale crystallinity, confirming the glassy nature of the residual carbon. Figure 2c inset shows images created by recording the amplitude of these peaks at each point on the mask. Mapping the amplitude to position recreates the images the original digital data. The D/G ratio among the mask region varied from 0.7 to 1.1, indicating disordered, amorphous carbon, further confirming the majority presence of glassy carbon. Electron dispersive x‐ray spectroscopy (EDS) of a Fresnel lens (Figure S1) reveals peaks of carbon, silicon, and oxygen (along with a trace amount of aluminum), consistent with the pyrolyzed carbon and the underlying fused silica substrate and rules out the presence of any higher atomic weight species. The spatial maps of the EDS data further confirm that the carbon is isolated to the patterned area.

#### 2.2.3 Atomic Force Microscopy of Pyrolyzed Films

An AFM scan of an opaque carbon film over approximately 10 µm × 10 µm region shows an RMS roughness of 0.453 nm (Figure S2). The data were leveled using a 3‐point level on the glass region. The RMS roughness of the carbon film (0.453 nm) is substantially lower than that of the underlying fused silica substrate (1.29 nm), indicating that the pyrolyzed film does not amplify substrate topography and may partially smooth high‐spatial‐frequency features. Because the AFM measurement reflects both the intrinsic carbon surface morphology and any remaining substrate‐induced topography, the reported roughness likely overestimates the intrinsic roughness of the carbon film. Use of optically polished substrates would therefore be expected to further reduce the measured surface roughness.

An optical microscope image of a carbon mask created from an image of micro‐electro‐mechanical (MEMS) gears is shown in Figure 2d. A high‐resolution AFM scan across the field indicated by the white box is shown in Figure 2e an individual profile denoted by the dotted line plot in 2e is plotted in Figure 2f. The topography along the gears indicates a continuous smooth transition across the grayscales in the image.

#### 2.2.4 Characterization of Light Transmission through GLARE Materials

Both positive‐ and negative‐tone photoresists transition from unexposed to fully exposed states across a finite exposure‐dose range. GLARE exploits this dose‐dependent behavior to fabricate structures with controlled resist heights. To implement a GLARE mask, the relationship between photoresin topography height and subsequent transmission intensity dose for an exposure wavelength must be established. The thickness of the carbon mask is a function of the carbon content of the photo‐resin and the exposed final height after the MPL write step. As this content varies (i.e., resists that are carbon rich should undergo less shrinkage, resulting in a thicker carbon mask for an equivalent MPL printed resist height), this relationship must be considered for every resist. To illustrate, we established the resist‐to‐transmission relationship for IP‐S that incurs significant volumetric shrinkage during pyrolysis. 1 mm^2^ plateaus were fabricated via MPL on fused silica with 240 nm step‐height variations between adjacent plateaus up to a total thickness of 6 µm. Additional isolated plateaus spanning resist thicknesses from 10–90 µm in 10 µm increments were fabricated, all characterized using stylus profilometry, and subsequently pyrolyzed. Figure 3a shows the false color map of the thinner carbon plateaus after pyrolysis. Plateau height was measured using white light interferometry (WLI) by averaging 10 columns over the plateau and is reported in each square. Figure S3 shows a computerized polarized light – differential interference contrast (C‐DIC) image of the mask with labeled columns. Figures S4–S8 shows the raw profilometer data along with the fitting of the profilometer using a step function.

**FIGURE 3:**
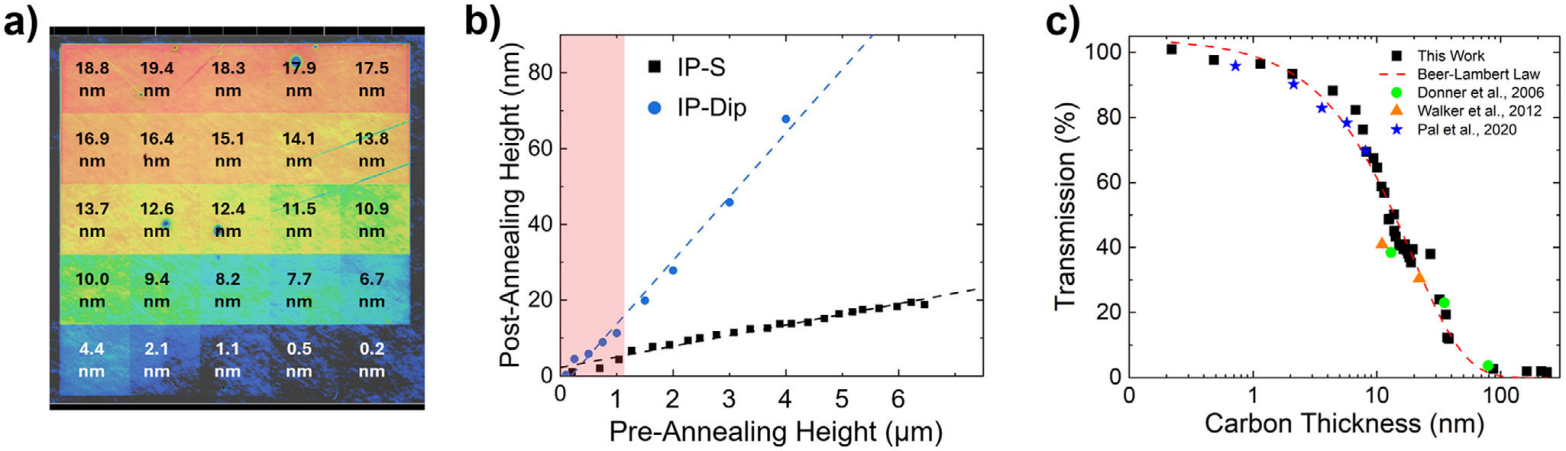
UV light transmission characteristics of GLARE masks. (a) Map of carbon plateau heights after pyrolysis. Each square is 1 × 1 mm. Inset numbers present the average plateau height for each square. (b) Plot of carbon plateau height vs initial photoresist height. The thinnest data points (pink region) are at the measurement limit for both the profilometer (resist) and white light interferometer (carbon). Linear fit parameters with standard errors: IP‐S slope = 2.8 ± 0.1 nm carbon/µm resist, intercept 2.3 ± 0.4; IP‐Dip slope = 16.8 ± 0.7 nm carbon/µm resist, intercept −3.1 ± 1.4. (c) Optical transmission vs. carbon thickness. Transmission for this work was measured at 365 nm; Donner et al. at 500 nm; Walker et al. at 365 nm; Pal et al. at 550 nm.

Figure 3b shows a graph of the carbon plateau height (WLI) vs. the resist height measured prior to pyrolysis from profilometry. Included in this plot is an analogous measurement for IP‐Dip (a negative‐tone photoresist from Nanoscribe GmbH), which indicates significantly less volumetric shrinkage compared to IP‐S. Data points in the pink region correspond to the three thinnest plateaus, which are near the limits of the measurement system. The dashed lines are linear fits to the IP‐S (R^2^ = 97.4%) and IP‐Dip (R^2^ = 98.7%) data, including the points in pink.

Optical transmission through the carbonized plateaus was measured using a fiber‐coupled xenon light source (AvaLight‐XE‐HP). The output of the xenon light source was collimated and focused to a spot < 1‐mm in diameter, then passed through each of the carbon plateaus. The transmission was collected into a fiber‐coupled spectrometer. Figure 3c contains a graph of the transmission at 365 nm versus measured carbon thickness. The transmission spans the range from approximately 98% (transparent) down to 0% transmission (opaque). The data were fitted with the Beer–Lambert law (red dotted line), consistent with exponential optical attenuation through the material. Notably, while the curves shown in Figure 3b are specific to the amount of shrinkage for IP‐S and IP‐Dip, the curve presented in Figure 3c is a fundamental property of the amorphous carbon. Other studies using different photoresists show an analogous dependence on optical transmission versus pyrolyzed carbon thickness [27, 28, 38]. Three of these studies are plotted in Figure 3c for comparison. The data for Donner et al. were recorded from pyrolysis of spin‐coated AZ P4330−RS films using 500 nm light, and 550 nm for Pal et al. For Walker, data at 365 nm were extracted for both thicknesses from their UV–vis curve from spin‐coated films of AZ 1518. The spectral response of pyrolyzed carbon is smooth and featureless, with a modest monotonic increase in transmission toward longer wavelengths (Figure S9). This wavelength dependence accounts for the slightly higher transmission values reported in the literature at 500 and 550 nm compared with those measured at 365 nm in this work, while supporting comparison across these wavelengths. Independently, the agreement between carbon transmission curves obtained from different photoresists in Figure 3c indicates that the relationship between carbon thickness and transmission is largely independent of the specific photoresist employed. The transition from transparent to opaque occurs within approximately 100 nm of carbon thickness, comparable to the chromium thickness used in conventional binary chrome‐on‐glass masks. Moreover, it implies that any lithographic approach for creating a 2.5D pattern (e.g., GSL, multilayer and thermal reflow lithography, MPL, maskless/single photon lithography, ink‐jet, etc.) can be used following shrinkage calibration of the photoresist (e.g., Figure 3b) and correlation to the carbon transmission curve (Figure 3c).

### 2.3 Microlens Array Fabrication Using GLARE

To examine the utility of this process, we designed a microlens array (MLA) consisting of lenslets with 80‐µm diameter on a grid spacing of 200 µm. Figure 4a shows a mathematically defined grayscale image of a lens that is input to fabricate an array using MPL of IP‐S resin with a peak height of 10 µm. Following pyrolysis conversion to a GLARE mask, a microscope slide was coated with positive‐tone AZ4330 photoresist, exposed through the GLARE mask and developed, yielding an array of lenslets on the surface. Exposure dose and develop times were adjusted to achieve complete exposure in the field outside the lenslets. Figure 4b shows an SEM image of a single lenslet and a profilometer scan of the surface of a single lens. Using the index of refraction of AZ 4330 (n ∼1.61), this lens should have a focal length of approximately 350 µm. To qualitatively demonstrate focusing by the fabricated array, the lenslets were illuminated with a 550 nm laser and the focal plane was imaged onto a camera as described schematically in Figure 4c. A relay lens system was used to accommodate the short focal length of the lenslets. Figure 4d is an optical microscope image of the array, while Figure 4e shows the resulting focal‐plane image, demonstrating focusing by the fabricated lenslets.

**FIGURE 4:**
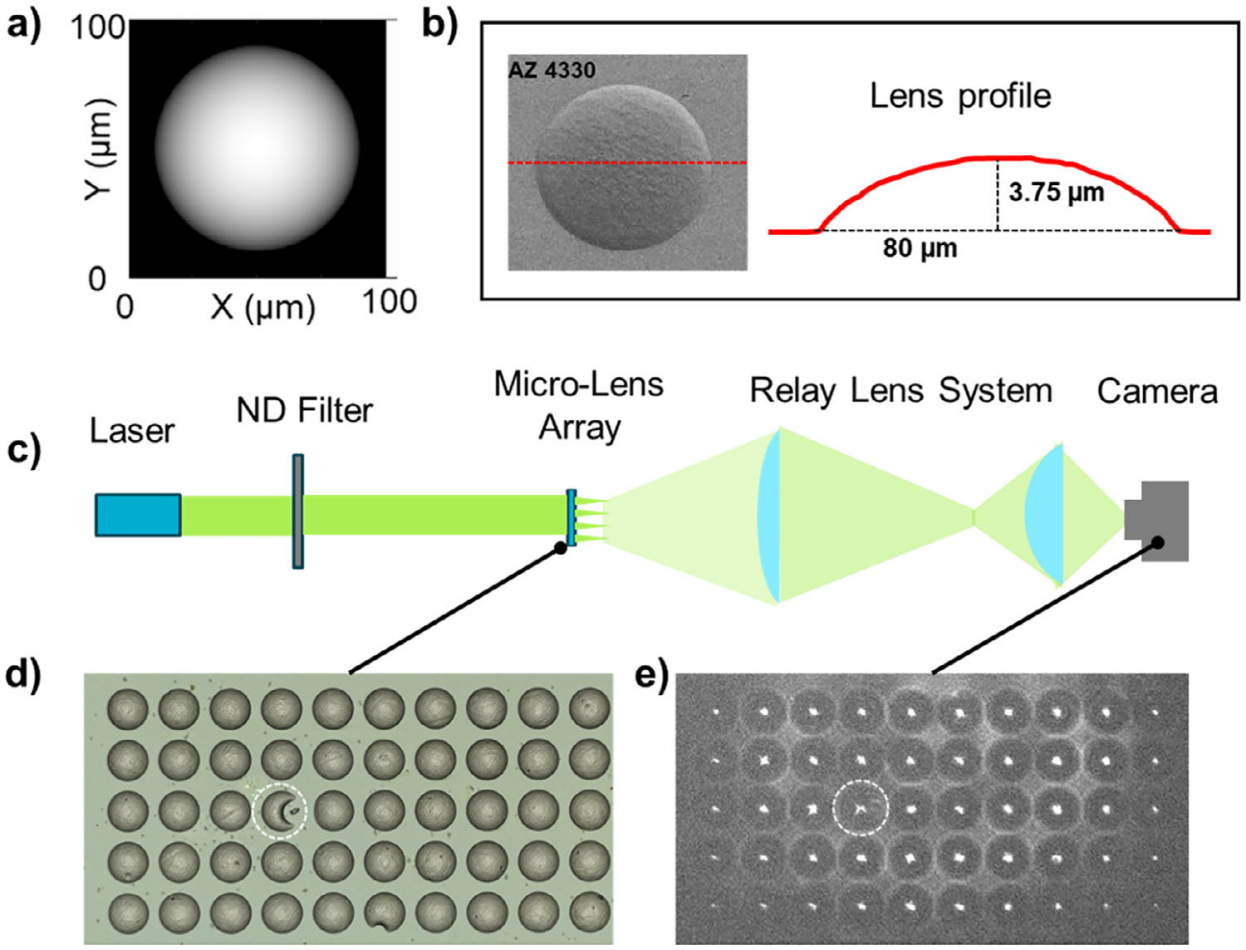
Fabrication of a microlens array using a GLARE mask. (a) A grayscale image of a lens in mapped from 0–255 in gray levels; (b) SEM image and profilometer scan of a single lenslet in the array after exposure through the grayscale mask. (c) Schematic of the optical set‐up used to capture the micro‐lens array focal plane; (d) Optical microscopy image of the MLA. (e) Captured image of the focal plane.

The darker circular halos around the focal spots are the outlines of the lenslets. The faint glow between the lenslets is representative of the illumination used. Each lenslet captures the incident field over the lens aperture and concentrates it at the focal spot, leading to the darker region. The carbon mask had a defect at one location (indicated by a dotted circle in Figure 4d), which shows up as the distorted focal spot in Figure 4e (indicated by the dotted circle in the image). Notably, the radius of curvature of the printed pattern can be adjusted by setting the 2.5D resist maximum height corresponding to the 255‐value in the grayscale. This demonstration highlights the scalability of the approach: although creation of the initial carbon mask requires a non‐trivial time investment for fabrication (areas surpassing square millimeters can require hours to 10s of hours) and a pyrolysis step (>30 hrs), the resulting reusable mask enables rapid grayscale replication of lenslet arrays in only seconds.

### 2.4 Mask Reusability

Mask reusability is a critical consideration for contact lithography because the mask is placed in direct contact with the photoresist‐coated wafer. Unlike projection lithography, where the reticle remains isolated within the optical system and avoids physical contact with the substrate, contact masks are routinely exposed to contamination from particles and photoresist residue. Consequently, repeated cleaning is required to maintain pattern fidelity, placing significant demands on the mechanical and chemical durability of the mask. The pyrolyzed carbon has a high Young's modulus (∼30 GPa [39]), strong adhesion to the fused silica blank, and is resistant to attack by solvents. Figure S10 shows two optical images of a grayscale retro‐reflector mask taken before and after a solvent clean step consisting of a 120‐s soak in acetone, followed by acetone/methanol/isopropyl alcohol rinses and finally compressed air drying. In the left image of Figure S10, taken after 10 exposures using this mask in contact lithography, multiple particles can be seen. After the solvent clean, the surface is clean of particles, and the mask shows no sign of physical or optical degradation.

## 3 Discussion

Despite decades of research, grayscale fabrication remains a niche approach to the creation of next‐generation 3D microstructures. Establishing a manufacturable grayscale lithography process requires precise mapping of subsequent pattern transfer process steps to the resist height profile after the grayscale step. Optimization of photoresist bake temperature and time, deviations in the linearity of exposure, development parameters, etch rates, and selectivity for pattern transfer into the underlying substrate may require multiple iterations for fabrication development. Depending on the final application, this iteration could potentially require alterations to the grayscale prescription of the mask as the most effective method to arrive at a satisfactory solution. Such iteration is expensive and significantly slower when the iteration loop requires rendering of mask data and shipment/fabrication at a commercial mask house. The current state‐of‐the‐art approach to the creation of grayscale masks, high energy beam sensitive (HEBS) glass, was invented in the 1990's. The HEBS‐glass approach uses an exotic chacogenide glass substrate which is irradiated with an electron beam to control the local transmission. Due to limited access to such materials and equipment, the technology is restricted to commercial mask houses. The approach outlined here uses widely available substrates such as quartz or fused silica, a commercial multi‐photon lithography platform, and a tube furnace. This route to production of grayscale masks is accessible to most university‐level fabs, drastically reducing the iteration cycle time for in‐house development.

While we have demonstrated the fundamental steps to the creation of grayscale masks in this work, several questions have yet to be resolved. The choice of pyrolysis temperature (900°C) was made to ensure removal of the majority of non‐carbon species in the pyrolyzed resist, however, there could be advantages in choosing a different temperature. It is possible the opacity of the pyrolyzed resist is achieved at a lower temperature prior to complete carbonization, or that enhanced opacity is achieved with higher temperature, moving the pyrolyzed resist further toward graphitization.

Choice of photoresist for the MPL step is also an important parameter. As we mentioned above, the carbon loading in the initial polymer affects the amount of shrinkage of the patterns. While IP‐S resist has shown > 98% vertical shrinkage, other negative resists such as SU‐8 can undergo far less change in height (40%–80%) depending on geometry and pyrolysis conditions [32, 34, 40]. Even though the patterns here show little distortion, the initial height of the photoresist could limit the range of achievable patterns. For example, tall resist patterns can be expected to impact our ability to place consecutive high‐contrast micrometer‐scale features next to one another. Thinner MPL resist alleviates this concern. Conversely, a resist which undergoes less shrinkage might yield an unacceptably large change in transmission for each voxel in MPL write z‐height. While the resultant glassy carbon has many advantages, it may also be possible to develop hybrid resists containing non‐volatile ceramic precursors, enabling conversion to polymer‐derived ceramics such as silicon carbide (SiC), silicon oxycarbide (SiOC), or silicon nitride (Si_3_N_4_) [19, 20]. Such compositions could expand the accessible range of optical transmission and absorption while providing enhanced thermal and chemical stability, potentially enabling durable transmission masks tailored to different exposure wavelengths.

Choice of mask substrate may also affect any distortion from the pyrolysis step. All the results presented here used fused silica blanks, however, quartz or sapphire blanks might provide more robustness to substrate distortion.

Finally, all the results presented here used multi‐photon lithography with a negative resist. Our experience with pyrolysis in a wide variety of other organic resists and epoxies, both positive and negative tone, indicates that other writing modalities could be used, such as single‐photon direct laser writing via a tool (e.g., Heidelberg DWL 66+) for creation of the blank master prior to pyrolysis, decreasing the mask writing time in cases where the resolution requirements of the pattern allow. For instance, in a reduction stepper system, the mask pattern is drawn at 4–5 times the size of the printed feature. In these systems, sub‐micron direct‐write resolution may not be necessary, allowing for faster mask write speeds.

We are hopeful that this approach will make true grayscale contact lithography more accessible to standard university and user facilities without requiring bespoke mask materials or commercial mask fabrication. Importantly, the technique is compatible with 2PP systems that are widespread amongst university and user facilities (e.g., Nanoscribe PPGT systems). Overall, reducing the time and expense required to iterate on the grayscale contact mask will accelerate exploration of the linear and non‐linear pattern transfer steps necessary to field next‐generation applications.

## 4 Materials and Methods

### 4.1 Reactants

The starting reagents were used as received: IP‐S and IP‐Dip photoresist (Nanoscribe), AZ4330 photoresist (MicroChemicals), 1″x1″ fused silica glass slides, silicon wafers.

#### 4.1.1 Creation of the Grayscale Mask

Grayscale images (png) were imported into the GrayScribe software used for Nanoscribe Quantum X printing, where the target area and height of structures is specified. This mode of MPL enables complex typographies to be printing in single layers by varying the exposure per voxel to modulate the structure height and achieve highly accurate 2.5D profiles. This proprietary mode of printing is not a requirement for generation of grayscale masks as typical layer‐by‐layer MPL (e.g., slice/hatch using DeScribe) can achieve similar results. Medium feature parameter presets for IP‐S fused silica were used, and the target thickness was in the eight‐ to ten‐micrometer range depending on the complexity of the mask and the desired height difference in greyscale levels.

The fused silica substrate was treated for adhesion promotion. Following 10 min air‐plasma treatment, the surface was incubated in ∼1:100 ratio (vol/vol) of 3‐(Trimethoxysilyl)propyl methacrylate: isopropyl alcohol (IPA) for >1 h, rinsed in IPA, and dried. Photoresist is deposited on the surface for a dip‐in configuration where a commercial two‐photon grayscale lithography printer (2GL; Quantum X Align, Nanoscribe) was used to write the 2.5D masks. Following exposure, the substrate was rinsed in propylene glycol monomethyl ether acetate (20 min) followed by IPA (2 min). Prior to further processing, the mask was thoroughly characterized using a profilometer.

### 4.2 Pyrolysis

The mask was pyrolyzed in a furnace at 900°C (ramp 1 K/min; held 30 min and cooled at 2 K/min to 25°C) under a reducing atmosphere of 3% hydrogen gas with argon to balance. This is to ensure the mask is converted into opaque carbon. Substantial shrinkage in height occurs, which is shown in Table S1 below.

### 4.3 Contact Lithography

Once the mask was fabricated, it was used in contact lithography on produce replicants at scale on a variety of substrates. For this work, wafer pieces were primarily used, however, glass and other substrates have been validated. A wafer was spin‐coated with AZ4330 photoresist according to manufacturer‐suggested procedures to a thickness between 3–5 micron. The i‐Line of a mercury lamp was then used to expose the wafer through the mask. Lastly, the wafer was developed in AZ400K 1:4, resulting in a patterned area on the wafer.

#### 4.3.1 Optical Characterization

At multiple stages in the fabrication process, images were captured using a Keyence VHX‐700 series optical microscope. Objective magnification ranged from 20x to 2500x depending on the size of the feature being captured. Lighting was primarily coaxial due to the relatively flat nature of the samples. When necessary, image stitching was done to capture high‐resolution images of the full sample. Focal variation was used to validate the stylus and laser profilometry.

### 4.4 Optical Profilometry

The masks before and after pyrolysis, as well as the lithographic transfer images, were scanned using both laser profilometry and white light interferometry (WLI). A Keyence VK‐X3100 was used to capture the 3D data with a 50x objective for laser profilometry, and a 10x objective was used for WLI. Stitched images were necessary for larger samples when doing laser profilometry. All data were processed and extracted in the VK MultiFile Analyzer application. Data were visualized in OriginLab.

### 4.5 Transmission Measurements

A fiber‐attached Avantes pulsed xenon light source (AvaLight‐XE‐HP) was collimated and focused down to an approximate 100 micrometer diameter spot size. The transmitted light was collected using a collection lens and a fiber collimator and coupled to a fiber‐attached Avantes Nexus spectrometer in the UV–vis range. The data were analyzed in Avasoft 8 and visualized in OriginLab.

### 4.6 Stylus Profilometry

Measurements were made on a Bruker Dektak XT with analysis done in OriginLab.

### 4.7 Raman Microscopy

Raman microscopy was performed using a 532 nm laser and 600 gr/mm grating. The objective was a 100 × 0.9NA Nikon objective. The step size was 1 um, scanning over an area of 235 × 180 um2. The integration time was 0.5 s per pixel for 1 accumulation. The laser power was kept at 5 mW and was determined not to affect the sample spectra over time. Spectra were fit by a sum of Lorentzian line shapes whose peak amplitudes were extracted to determine D‐ and G‐peak intensities.

## Author Contributions

T.D.C. contributed Optical characterization and writing. G.C.G. contributed Optical characterization and writing. Z.T.P. contributed spatially resolved Raman analysis. T.T‐T. contributed spatially resolved Raman analysis. R.R. contributed electron‐dispersive spectra and analysis. E.R.A. contributed pyrolysis and thermal processing. M. R. contributed AFM collection and analysis. J.D.B. contributed pyrolysis and thermal processing. B.J.K. contributed two‐photon lithography, experiment conceptualization, and writing. D.B.B. contributed experiment conceptualization, mask design, photolithography, and writing.

## Conflicts of Interest

The authors declare no conflicts of interest.

## Supporting information




**Supporting File**: advs78394‐sup‐0001‐SuppMat.docx.

## Data Availability

The data that support the findings of this study are available on request from the corresponding author. The data are not publicly available due to privacy or ethical restrictions.
