# Supplementary material for: Pyrolyzed Photoresist as a Continuous‐Tone Mask for Grayscale Lithography
Source: Adv Sci (Weinh). 2026 Oct 18:e78394. Online ahead of print. doi: 10.1002/advs.78394 (PMC13677049; doi:10.1002/advs.78394)
Supplement: Supplementary file 1 [file ADVS-9999-e78394-s001.docx]

Supporting Information for:

Pyrolyzed Photoresist as a Continuous-Tone Mask for Grayscale Lithography

**Authors.**

Tielyr D. Creason, Gavin C. Gee, Zachary T. Piontkowski, Toai Ton-That, Robert Reyna, Edward R. Arata, Mason Risley, Jacob D. Boissiere, Bryan Kaehr*, and D. Bruce Burckel*

Sandia National Laboratories, Albuquerque, NM, 87185, USA

E-mail: [dbburck@sandia.gov](mailto:dbburck@sandia.gov); bjkaehr@sandia.gov

Keywords: (lithography, gray scale, pyrolyzed carbon)

**I. EXPERIMENTAL DETAILS.**

**1. Reactants.** The starting reagents were used as received: IP-S and IP-Dip photoresist (Nanoscribe), AZ4330 photoresist (MicroChemicals), 1”x1” fused silica glass slides, and 150-mm silicon wafers.

**2. Creation of the Grayscale Mask.**

**2.1 Grayscale Data.** A source digital image was converted to 255 levels of gray and input into GrayScribeX (Nanoscribe). Medium feature parameter presets for IP-S fused silica were used and the target thickness was in the eight to ten micrometer range depending on the complexity of the mask and the desired height difference in greyscale levels.

**2.2 Writing of the Mask.** The fused silica substrate was treated for adhesion promotion. Following 10 minutes air-plasma treatment, the surface in incubated in ~ 1:100 ratio (vol/vol) of 3-(Trimethoxysilyl)propyl methacrylate: isopropyl alcohol (IPA) for >1 hour, rinsed in IPA and dried. Photoresist is deposited on the surface for dip-in configuration where a commercial two-photon grayscale lithography printer (2GL; Quantum X Align, Nanoscribe) was used to write the 2.5D masks. Following exposure, the substrate is rinsed in propylene glycol monomethyl ether acetate (20 min) followed by IPA (2 min). Prior to further processing the mask was thoroughly characterized.

**2.3 Pyrolyzing of the Mask.** A The mask was pyrolyzed in a furnace at 900 ℃ (ramp 1 K/min; held 30 min and cooled at 2 K/min to 25℃) under a reducing atmosphere of 3% hydrogen gas with argon to balance. This is to ensure the mask is converted into opaque carbon. Substantial shrinkage in height occurs, which is shown in Table S1 below.

**3 Lithographic Transfer.** Once the mask described in section 2, was fabricated it was used in a lithographic transfer process to produce replicants at scale on a variety of substrates. For this work wafer pieces were primarily used, however glass and other substrates have been validated. A wafer was spin coated with AZ4330 photoresist according to manufacturer suggested procedures to a thickness between 3-5 micron. A commercial contact aligner (Suss MA-6) with mercury i-Line (365 nm) was then used to expose the wafer through the mask. The exposure dose was adjusted to achieve desired exposure results, with exposures typically in the 8-15 second range with exposure energy density of 20mJ/cm^2. Lastly the wafer was rinsed in DI water to remove exposed photoresist, resulting in a patterned area on the wafer.

**4 Optical Characterization.**

**4.1 Optical Images.** At multiple stages in the fabrication process images were captured using a Keyence VHX-700 series optical microscope. Objective magnification ranged from 20x to 2500x depending on the size of feature being captured. Lighting was primarily coaxial due to the relatively flat nature of the samples. When necessary, image stitching was done to capture high resolution images of the full sample. Focal variation was used to validate the stylus and laser profilometry.

**4.2 Optical Profilometry.** The masks before and after pyrolysis, as well as the lithographic transfer images were scanned using both laser profilometry and white light interferometry (WLI). A Keyence VK-X3100 was used to capture the 3D data with a 50x objective for laser profilometry and a 10x objective used for WLI. Stitched images were necessary for larger sample when doing laser profilometry. All data was processed and extracted in the VK MultiFile Analyzer application. Data was visualized in OriginLab.

**4.3 Transmission Measurements.** To establish the thickness dependent transmission curve a simple optical measurement station was constructed. A fiber attached Avantes pulsed xenon light source was collimated and focused down to an approximate 100-micron diameter spot size. The light was collected on the other side of a pyrolyzed mask using a collection lens and a fiber collimator. The light was collected using a fiber attached Avantes Nexus spectrometer in the UV-Vis range. The data was analyzed in Avasoft 8 and visualized in OriginLab.

**5 Stylus Profilometry.** The masks prior to pyrolysis and the lithographic transfer images were also measured using stylus profilometry to cross validate optical profilometer measurements. Measurements were made on a Bruker Dektak XT with analysis done in OriginLab.

**II. SUPPLEMENTAL INFORMATION.**

**Table S1** Shrinkage and deformation of structures during pyrolysis

| **Structure** | **Total Volume (μm^3^**) | **Surface Area (μm^2^)** | **Average Height (μm)** | **X-width (μm)** | **Y-width (μm)** | **Pyrolysis Volumetric Shrink** |
| --- | --- | --- | --- | --- | --- | --- |
| Eye Initial | 237342.97 | 71656.23 | 6.564 | 241.24 | 179.32 |  |
| Eye Post-pyrolysis | 463.60 | 48897.83 | 0.009 | 246.91 | 185.54 | 99.80% |
| Rings Initial | 3915232.89 | 997703.04 | 4.063 | 1000.62 | 997.21 |  |
| Rings Post-pyrolysis | 8036.11 | 1018958.65 | 0.008 | 1035.10 | 1036.99 | 99.79% |

**Figure S1.** (Top panel) Electron dispersive x-ray spectroscopy data showing carbon, oxygen, aluminum and silicon peaks; (inset) optical microscope transmission image of Fresnel lens. (bottom panels). Elemental spatial maps from EDS data indicate carbon is confined to the written pattern area.

**Figure S2.** AFM scan of an opaque carbon region with 0.453 nm RMS roughness.

**Figure S3.** Tiered sample CDIC image from laser profilometry scans with column label

**Figure S4.** (A) Fitted profilometry pre-pyrolysis of column one in the tiered structures, (B) post-pyrolysis fitted heights.

**Figure S5.** (A) Fitted profilometry pre-pyrolysis of column two in the tiered structures, (B) post-pyrolysis fitted heights.

**Figure S6.** (A) Fitted profilometry pre-pyrolysis of column three in the tiered structures, (B) post-pyrolysis fitted heights.

**Figure S7.** (A) Fitted profilometry pre-pyrolysis of column four in the tiered structures, (B) post-pyrolysis fitted heights.

**Figure S8.** (A) Fitted profilometry pre-pyrolysis of column five in the tiered structures, (B) post-pyrolysis fitted heights.

**Figure S9.** Optical microscope images of a carbon mask before and after solvent cleaning. Two particles (identified by the blue arrows) present before cleaning are removed after cleaning, with no visible degradation of the mask pattern.

**Figure S10.** Test structures fabricated with the Nanoscribe Photonic Professional GT2 printer (PPGT2; IP-S photoresist, 25X objective) with a designed maximum height of 10 µm prior to (a) and following pyrolysis (b). Insets show a profilometry line scan across the test structure indicated by the red line. Scale bars, 100 µm.
